# Acute Coronary Syndrome in the Older Patient

**DOI:** 10.3390/jcm10184132

**Published:** 2021-09-14

**Authors:** Sergio García-Blas, Alberto Cordero, Pablo Diez-Villanueva, Maria Martinez-Avial, Ana Ayesta, Albert Ariza-Solé, Gemma Mateus-Porta, Manuel Martínez-Sellés, David Escribano, Ana Gabaldon-Perez, Vicente Bodi, Clara Bonanad

**Affiliations:** 1 Cardiology Department, Hospital Clínico Universitario de Valencia, INCLIVA Biomedical Research Institute, University of Valencia, 46010 Valencia, Spain; sergiogarciablas@gmail.com (S.G.-B.); anagabaldonperez@gmail.com (A.G.-P.); vicentbodi@hotmail.com (V.B.); 2 Centro de Investigación Biomédica en Red de Enfermedades Cardiovasculares (CIBERCV), 28029 Madrid, Spain; acorderofort@gmail.com (A.C.); mmselles@secardiologia.es (M.M.-S.); d.escribanoalarcon@gmail.com (D.E.); 3 Cardiology Department, Hospital Universitario de San Juan, 03550 Alicante, Spain; 4 Cardiology Department, Hospital Universitario de La Princesa, 28006 Madrid, Spain; pablo_diez_villanueva@hotmail.com (P.D.-V.); mariam-avial@hotmail.es (M.M.-A.); 5 Cardiology Department, Hospital Central de Asturias, 33011 Oviedo, Spain; ana.ayestalopez@gmail.com; 6 Cardiology Department, Hospital Universitari de Bellvitge, L’Hospitalet de Llobregat, 08907 Barcelona, Spain; aariza@bellvitgehospital.cat (A.A.-S.); gemmamateus@hotmail.com (G.M.-P.); 7 Cardiology Department, Hospital Universitario Gregorio Marañón, Universidad Europea, Universidad Complutense, 28007 Madrid, Spain

**Keywords:** elderly, acute coronary syndrome, myocardial infarction

## Abstract

Coronary artery disease is one of the leading causes of morbidity and mortality, and its prevalence increases with age. The growing number of older patients and their differential characteristics make its management a challenge in clinical practice. The aim of this review is to summarize the state-of-the-art in diagnosis and treatment of acute coronary syndromes in this subgroup of patients. This comprises peculiarities of ST-segment elevation myocardial infarction (STEMI) management, updated evidence of non-STEMI therapeutic strategies, individualization of antiplatelet treatment (weighting ischemic and hemorrhagic risks), as well as assessment of geriatric conditions and ethical issues in decision making.

## 1. Introduction

Coronary artery disease is one of the leading causes of mortality and morbidity worldwide, and its prevalence increases with age [1,2,3]. The lengthening of life expectancy has caused the proportion of older patients admitted for acute coronary syndrome (ACS) to rise significantly, with one in every three patients presenting with ACS being over 75 years old [4].

The older patient has clinical peculiarities that pose a higher risk in this setting, such as comorbidities and geriatric syndromes. Diagnosis and therapeutic approach are also more challenging in this age group due to a higher prevalence of atypical features and an increased vulnerability to side effects and complications [5,6]. Moreover, older patients are often underrepresented in large clinical trials, and there is a paucity of specific evidence.

The aim of this review is to summarize the latest knowledge about some key points of ACS management in older patients.

## 2. Diagnostic Approach in the Older Patient

The ACS diagnostic pathway is the same as recommended for the general population. However, some peculiarities and challenges should be noted. Atypical symptoms are more frequent, and, together with communication difficulties, may lead to delays or misdiagnoses [7]. Another source of diagnostic uncertainties is the higher frequency of baseline electrocardiogram changes, such as bundle branch block or pacemaker rhythm [7].

Even more challenging may be the interpretation of troponin elevations in the older patient. Elevated basal troponin levels have been described, and it is known that age > 60 years is associated to higher 99th percentile upper reference limit [8,9,10]. Boeddinghaus et al. analyzed the diagnostic performance of the 0/1 h algorithm recommended by the European Society of Cardiology in three age groups (<55 years, young; 55–70 years, middle age; ≥70 years, old) [11]. They found similar rule-out safety (sensitivity) while decreased rule-in accuracy (specificity), 93% for young, 80% for middle-age, and 55% for old (*p* < 0.001). Using slightly higher cut-off troponin concentrations specific for older patients resulted in increased specificity while maintaining a high sensitivity, especially when using troponin-I [11]. However, the main consensus documents and clinical practice guidelines do not currently support the use of age-specific thresholds [12,13]. Further research is warranted to clarify the best approach.

## 3. ST-Segment Elevation Myocardial Infarction (STEMI) Patients

Emergent reperfusion, especially primary percutaneous coronary intervention (PCI), is the standard of care in STEMI, and its widespread use has improved both short and long-term prognosis [14,15]. This applies equally to the older patient, but some peculiarities must be noted.

Regarding diagnosis, atypical clinical presentation and communication difficulties (derived from confusional states or cognitive impairment) are more frequent in the elderly and may delay diagnosis [16,17]. Baseline electrocardiogram alterations may also make diagnosis difficult in older patients, since some findings, including history of previous myocardial infarction, pacemaker stimulation, or the presence of left bundle branch block, are common [14,16].

**Figure 1:**
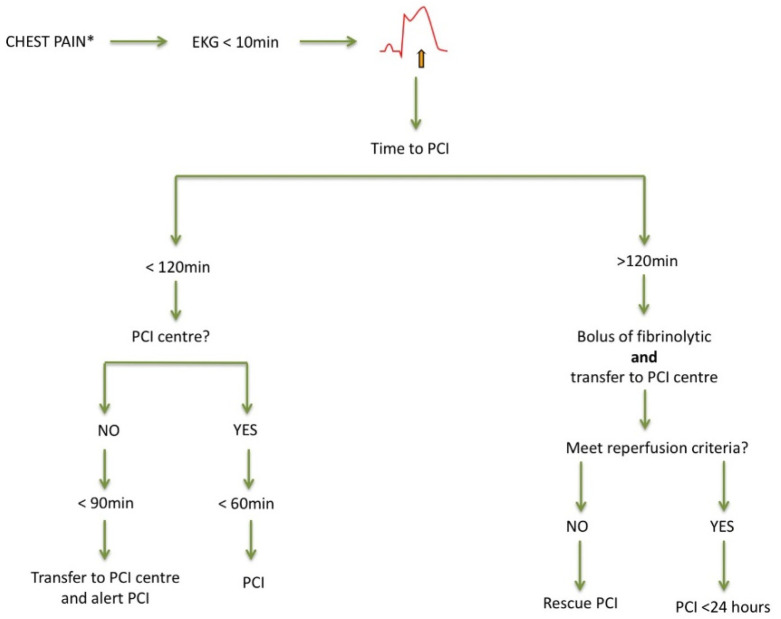
Management of acute ST-segment elevation myocardial infarction. Adapted from [16]. EKG: electrocardiogram; PCI: percutaneous coronary intervention. * Atypical presentation is common.

According to current guidelines, primary PCI performed <120 min since the first medical contact is the treatment of choice, irrespective of age. PCI is superior to fibrinolysis in reducing mortality, reinfarction, or stroke [18]. It is recommended to use radial access and stenting with new-generation drug-eluting stents, since this strategy is associated with fewer events [18,19]. Otherwise, and in the absence of contraindications, fibrinolytic therapy is recommended, always within the first 12 h since symptom onset, and providing the highest benefit when administered within the first two hours. Afterwards, the patient must be transferred to a center with a PCI-capable facility (Figure 1) [18]. Thus, if reperfusion is not achieved (ST-segment resolution < 50% within 60–90 min of fibrinolytic administration) or in the presence of persistent chest pain, worsening ischemia, or hemodynamic or electrical instability, rescue PCI is indicated. In other cases, routine early (2–24 h after fibrinolysis) PCI is indicated. Age should not be considered a contraindication to fibrinolysis, since several studies including elderly patients with STEMI receiving this therapy did not find significant differences in the number of major bleedings requiring transfusion compared to PCI or even no reperfusion [20]. On the other hand, the incidence of intracranial bleeding increases with age. Nevertheless, in the STREAM trial, the risk of intracranial hemorrhage was reduced after adjusting the fibrinolytic dose of tenecteplase to 50% in patients older than 75 years of age [21].

Despite current evidence and recommendations, older patients are still less likely to receive reperfusion treatment when compared with their younger counterparts [3,16,19,22]. Efforts should be made to improve this picture, as invasive strategies in STEMI associate greater survival in elderly patients, and there is no upper age limit for urgent reperfusion [3,19,22,23].

Regarding prognostic medication in the elderly, current recommendations are not different from those made in younger patients. They improve prognosis [15], though titration may often be slower [14,18]. Moreover, elderly patients should participate in a complete rehabilitation program whenever possible, adapted to age conditions, and addressing comorbidities and geriatric syndromes, which in turn improves both prognosis and quality of life [18]. Unfortunately, these patients are, by far, the least enrolled in these programs [24].

## 4. Non-STEMI (NSTEMI) Patients: Invasive Versus Conservative Treatment?

Balancing the benefit/risk of harm is crucial in elderly patients with ACS because they have higher risk of mortality but, also, higher risk of bleeding or other side effects of currently recommended treatments. Compared to younger patients, older patients are admitted more frequently with NSTEMI and medical treatments or revascularization are commonly underused [25,26]. Current NSTEMI guidelines clearly state that interventional procedures for revascularization should be applied at any age [13]. Nonetheless, age is frequently reported as the variable most closely related to lower revascularization rates [27]. Of all the reasons postulated for the underuse of revascularization, the leading limitations might be the excess of bleeding complications or the lack of long-term benefit [28]. Bleeding complications have certainly increased in recent decades due to the incorporation of more potent antiplatelet and anticoagulant strategies [29]. In contrast, vascular access complications have decreased substantially with the generalization of radial access, especially in the elderly and high-bleeding risk patients [30,31]. The largest clinical trial involving elderly patients with NSTEMI, or unstable angina, was the After Eighty study that included 457 patients who were randomized to either an invasive or conservative treatment strategy [32]. After 1.5 years of follow-up, the invasive strategy was superior to a conservative strategy in reducing the primary endpoint, namely, a composite of myocardial infarction, urgent revascularization, stroke, or death. The primary endpoint was reduced by 47% using the invasive strategy (hazard ratio 0.53, 95% CI 0.41–0.69; *p* = 0.0001), although the benefit was lower in patients aged >85, and no differences in terms of bleeding complications were noted. It should be noted that in this trial, a strict conservative strategy was applied instead of a selective invasive strategy, with no coronary angiography performed in any patient assigned to this group. In 2020, the SENIOR-NSTEMI study was published, which was an observational study with 1976 NSTEMI patients aged >80 [33]. After propensity score matching, it showed that revascularization within the first 3 days of admission was associated to 32% reduction in all-cause mortality (hazard ratio: 0.68, 95% CI 0.55–0.84). This study had several limitations because it did not assess the effect of heart failure of index hospitalization on post-discharge prognosis, and heart failure incidence was not assessed taking all-cause mortality as a competing event [34,35].

Lastly, revascularization procedures could also be discussed. The use of drug-eluting stents has been generalized in percutaneous revascularization, and several trials have demonstrated their superiority compared to bare-metal stents also in the elderly [36]. Polymer-free drug-eluting stents have demonstrated to have a very low risk of stent thrombosis with short-term dual antiplatelet treatments [37], and this has also been demonstrated in second and third generations of drug-eluting stents [38]. Another important aspect is the relevance of the complete revascularization in this age group. Agra-Bermejo et al. observed in a propensity-score analysis of an observational cohort a long-term benefit in terms of mortality of complete vs. culprit-only revascularization in older patients with NSTEMI [39]. However, other studies showed controversial results [40,41]. Further randomized evidence is warranted to clarify the better strategy regarding complete revascularization in the older patient.

In conclusion, an invasive strategy with currently available technologies (radial access and newer generation drug-eluting stents) is safe and effective to improve outcomes in elderly patients with NSTEMI and, therefore, it should not be denied in this patient group.

## 5. Antithrombotic Treatment: Antiplatelets and Anticoagulants

### 5.1. Bleeding Risk in Elderly Patients with Acute Coronary Syndromes

Risk prediction in elderly patients with ACS is challenging, since these patients are usually excluded from clinical trials and evidence about their optimal risk prediction and management is scarce [42]. Conditions such as diabetes mellitus, renal dysfunction, and anemia are more frequent in elderly ACS population. All such conditions are associated with higher rates of ischemic and hemorrhagic complications [43,44,45]. However, risk prediction in the older patient remains still a challenge. Current guidelines recommend estimating ischemic (class IIa) and hemorrhagic risks (class Iib) when addressing treatment approach in the setting of an ACS [13]. However, regular bleeding risk scores have proved limited value in older patients with ACS, which is probably because they are developed from clinical trials where they are underrepresented [46]. The use of PRECISE-DAPT score is recommended to guide dual antiplatelet therapy duration after an ACS, suggesting a more conservative antithrombotic approach in patients with a score ≥ 25 [47]. It is worth noting that an age of 75 years or more gives 12 points on the scale. Therefore, most older patients will have PRECISE-DAPT values ≥ 25, i.e., they are classified as high bleeding risk solely because of age. Some studies suggest that this cut-off point might not be the most optimal approach in people of advanced age, even more so considering their usual higher thrombotic risk (higher prevalence of diabetes, peripheral vascular disease, extensive coronary artery disease, etc.) [48]. The use of adjusted thresholds in the older patient may probably be a more rational approach to predict bleeding risk.

### 5.2. Antiplatelet

Antithrombotic therapy is a cornerstone of the treatment in ACS both with and without an invasive approach. The decision regarding P2Y12 inhibitor, drug dosing, time initiation, and duration depends on individual clinical judgment driven by the patient’s ischemic and bleeding risk. Current guidelines recommend treatment with acetylsalicylic acid and a potent P2Y12 receptor inhibitor (prasugrel or ticagrelor) over 12 months [13]. Clopidogrel, which exhibits a weaker and more variable platelet inhibition, is recommended when prasugrel and ticagrelor cannot be administrated due to bleeding risk or contraindications [49,50]. This recommendation is based on the data from the PLATO (PLATelet inhibition and patient Outcomes) [51] and TRITON-TIMI 38 (Prasugrel versus Clopidogrel in Patients with Acute Coronary Syndromes) trials [52], which showed the superiority of ticagrelor and prasugrel compared to clopidogrel by means of reducing cardiovascular death, myocardial infarction, and stroke. However, both trials included mostly younger patients without comorbidities. Probably due to scarce and conflicting published data, clopidogrel is the most widely used P2Y12 blocker in older patients in routine clinical practice [53].

In a subgroup analysis from the PLATO trial, no increase in overall major bleeding with ticagrelor versus clopidogrel was observed in patients aged ≥ 75 years, suggesting that the antithrombotic benefits of ticagrelor also can be applied to older individuals [54]. In addition, some authors have described a low incidence of post-discharge bleeding in carefully selected very elderly ACS patients treated with ticagrelor in routine clinical practice [55]. In contrast, a recent observational analysis from the SWEDEHEART registry assessed the effects of ticagrelor versus clopidogrel among 14,005 consecutive patients age 80 years or older with myocardial infarction. In the main analysis, ticagrelor was associated with a lower risk of myocardial infarction and stroke but also with an increased risk of death and bleeding compared to clopidogrel [56]. The authors stressed the need for a specific randomized trial of P2Y12 inhibitors in older patients with ACS.

On the other hand, the TRITON-TIMI 38 trial randomized 13,608 patients with ACS with scheduled PCI to receive clopidogrel or prasugrel for 6 to 15 months. In the subgroup analysis, no clinical benefit of prasugrel was observed in patients > 75 years due to their higher rates of bleeding (HR 0.99; 95% CI 0.81–1.21; *p* = 0.92) [52]. Moreover, reducing the titration of prasugrel to 5 mg based on a platelet function test did not improve the clinical outcome of patients >75 years after an ACS [57].

More recently, the POPular AGE (Ticagrelor or Prasugrel Versus Clopidogrel in Elderly Patients with an Acute Coronary Syndrome and a High Bleeding Risk: Optimization of Antiplatelet Treatment in High-risk Elderly) trial randomized 1003 patients aged 70 years old or older with non-ST elevation acute coronary syndrome to receive clopidogrel versus ticagrelor or prasugrel for 12 months. The authors described a lower bleeding rate with clopidogrel (HR 0.71; 95% CI 0.54–0.94; *p* = 0.03) without an increase in the combined endpoint of all cause of death, myocardial infarction, stroke, and bleeding. However, some significant issues with this study were observed, such as a high rate of discontinuation of the study drug (47% and 22% in ticagrelor and clopidogrel arms, respectively) [58]. The authors concluded that clopidogrel could be an alternative to potent P2Y12 inhibitors, especially for elderly patients with a higher bleeding risk.

Nonetheless, results from the START-ANTIPLATELET Registry showed that in high bleeding risk patients, the duration of the dual antiplatelet therapy and not the second antiplatelet chosen (clopidogrel or ticagrelor) was associated with the bleeding risk and net clinical benefit at 1 year [59].

As a result of these conflicting results, optimal antithrombotic therapy in elderly patients is still under debate, and recommendations from current guidelines should be interpreted with caution. Well designed, randomized clinical trials are needed to test the efficacy and safety of antiplatelet drugs in high-risk elderly patients.

### 5.3. Anticoagulants

The elderly population is characterized by extreme heterogeneity, thus precluding general recommendations in this group in routine clinical practice. In the field of periinterventional anticoagulant treatment in ACS, unfractionated heparins are preferred because of their predictable dose–effect relationship. Especially in elderly patients, anticoagulant doses must be adjusted to body weight and renal function to reduce the risk of PCI-related bleeding. Moreover, longer treatment duration is associated with a higher incidence of bleeding [56].

On the other hand, some conditions that require oral anticoagulation (atrial fibrillation, stroke) are especially common at older ages. In non-ST elevation acute coronary syndrome patients, evidence on the management of patients requiring long-term oral anticoagulant therapy is derived from subgroups of randomized controlled trials [46]. Overall, in patients with non-valvular atrial fibrillation, the evidence supports the use of direct oral anticoagulants over vitamin K antagonists in terms of bleeding risk [60,61]. The recommended strategy is a short period of triple therapy (i.e., dual antiplatelet therapy with clopidogrel and anticoagulation with a direct oral anticoagulant), followed by direct oral anticoagulant and single antiplatelet therapy (preferably clopidogrel) up to 12 months. This strategy should be individualized, with a longer period of triple therapy (up to one month) in patients at higher ischemic risk or shorter (1 week) in patients at high bleeding risk [46].

Given the lack of information in elderly patients, decisions should be taken according to individual risk profile and burden of comorbidities. Furthermore, specific randomized trials are needed to achieve a better understanding of the impact of anticoagulation strategy on the clinical outcomes of elderly patients with ACS.

## 6. Geriatric Conditions: Frailty and Comorbidity

Most evidence on ACS in the older patient focuses on age. However, age itself does not accurately reflect the patient’s status, as other characteristics such as comorbidities and geriatric syndromes (frailty, disability, cognitive impairment, etc.) are the key determinants of patient’s health and vulnerability beyond age [62,63].

Frailty is defined as a clinical syndrome in which there is an increased vulnerability of adverse events (disability, need for hospitalizations, institutionalization, and death) in in situations of endogenous or exogenous stressors [64,65,66,67,68]. It is present in about 10% of patients > 65 years and 25–50% of those > 85 admitted with ACS, although these numbers may vary depending on the definition applied [69].

Frailty is a well-stablished independent predictor of short and long-term mortality after an ACS [70,71,72,73,74,75,76,77,78]. In fact, there are data that suggest that it is the main prognostic determinant within geriatric syndromes [72,77,79]. Several frailty scales are available and validated in this setting, and even isolated components of them (mainly gait speed) are valuable for risk prediction [74,76,80]. Despite this high risk, frail patients are less likely to receive coronary angiography, PCI, complete revascularization, or even evidence-based medical therapy [69]. This conservative approach (or rather, undertreatment) is based merely on physician perception and not in clinical evidence, since to date, there are no data clarifying the best strategy for frailty assessment in ACS and the most appropriate management when present. In the Spanish LONGEVO registry, older patients (≥80 years) with ACS treated conservatively showed a higher rate of cardiac death, reinfarction, or new revascularization at six months, but only if non-frail, whereas no significant association was observed in frail patients [81]. Other observational data suggest the opposite, finding a better outcome in frail patients with non-ST elevation acute coronary syndrome when treated with PCI [76]. An ongoing clinical trial (The Invasive and Conservative Strategies in Elderly Frail Patients with Non-STEMI, NCT03208153) that randomizes frail patients older than 70 years to a routine invasive vs. conservative (selective invasive) strategy will shed light on this matter [82].

A possible explanation for choosing a conservative approach in frail patients with ACS is perceiving greater predisposition to complications. However, there is no strong evidence supporting this assumption. Controversial results have been published regarding bleeding risk in frail patients. Alonso et al. found frailty as an independent predictor of major bleeding in older patients (≥75 years) with an ACS [83]. On the other hand, a substudy of the TRILOGY ACS (Targeted Platelet Inhibition to Clarify the Optimal Strategy to Medically Manage Acute Coronary Syndromes) trial showed a lack of relationship between frailty and bleeding at 30 months in patients with ACS aged 65 years or more [84]. In the same line, a Spanish multicenter registry (LONGEVO) of ACS patients ≥ 80 years found that neither frailty nor other geriatric syndromes had an adequate predictive capacity regarding hemorrhagic events in this setting [42]. Differences in patient baseline characteristics and definitions of both frailty and bleeding events may explain these discrepancies. In any case, the evaluation and management of these patients should be individualized without denying invasive therapies based merely on the presence of frailty.

To date, there is no specific evidence on the approach to frailty in these patients nor on whether we can modify their status or prognosis through some type of intervention. Sanchis et al. randomized 150 survivors after an acute myocardial infarction ≥ 70 years and with pre-frailty or frailty, to a 3-month exercise program, under physiotherapist supervision, followed by an independent home-based program versus standard care [85]. They found that frailty status improved significantly in the subgroup that participated in the program. However, only 60% of patients randomized to intervention completed the program, and the benefit observed was mainly at 3 months and it was attenuated at one year. Moreover, no differences in clinical events were observed [85]. These results highlight the challenge in implementing a cardiac rehabilitation program in the older patient but also the potential benefit if completed.

Cognitive impairment may hinder diagnosis, decrease therapeutic compliance, and worsen quality of life. Some evidence supports the prognostic value of cognitive impairment on top of frailty in older patients after an ACS [86]. Early detection of mild cognitive impairment can help optimize treatment at discharge to improve compliance and decrease complications.

Body mass index is proposed to have an impact on prognosis in ACS, with the so-called obesity paradox. However, when adjusting for age and other clinical conditions, body mass index seems to lose its prognostic value [87].

Age is also associated with a higher burden of comorbidities, which turns out to have a marked prognostic impact [88]. Clinical entities such as vascular disease, diabetes, and chronic kidney disease are associated with more severe coronary artery disease. On the other hand, heart failure, pneumological and oncological disorders, and severe liver disease, among others, can condition the management of ACS.

Different comorbidity indices have been created to express the overall burden of comorbidity in a reproducible way. The Charlson index is the most widely used, and it has validated prognostic value in this setting [89]. However, it was developed based on a population substantially different from ACS patients, and it include some diseases infrequent in the elderly, while other conditions of potential interest in ACS are not considered. More simplified indexes (such as a score including renal failure, anemia, diabetes, peripheral artery disease, cerebrovascular disease, and chronic lung disease) have proven a similar performance for risk assessment after ACS [90].

Most studies show a firm relationship between the degree of comorbidity and complications associated with ACS [89]. Researchers have also reported an association between Charlson index and readmissions after PCI [91]. In the LONGEVO registry, comorbidity was the only component of the geriatric evaluation that was associated with the appearance of bleeding [42]. These worse clinical outcomes are probably related to the fact that some of the components of the Charlson Index are well-established predictors of bleeding (neoplasia, kidney failure, liver disease).

There is no clear evidence of the impact of comorbidities on the benefit of invasive therapy in ACS. In an observational study, Chang et al. found a lack of benefit of invasive strategy in the subgroup with higher comorbidity burden (i.e., Charlson Index ≥ 4). On the other hand, a retrospective study with >7200 patients aged ≥ 70 years from 11 Spanish ACS registries analyzed the impact of the six most common comorbidities (diabetes, peripheral artery disease, cerebrovascular disease, chronic pulmonary disease, renal failure, and anemia) on revascularization, finding that revascularization reduced 1-year mortality despite the presence of comorbidities [35]. The MOSCA trial randomized routine invasive vs. conservative strategy in comorbid older patients with non-STEMI. The invasive strategy did not improve outcomes in terms of mortality or ischemic events at long term follow-up, although a significant benefit was observed at 3 months in a non-prespecified subanalysis [92]. Further investigation is warranted to clarify the best management of comorbidities in ACS. Table 1 summarize some of the key evidence on ACS in the older patient.

## 7. Ethics Conditions

In older patients with ACS, ethical considerations regarding management and treatment are common, especially when deciding invasive vs. conservative treatment, type of drug therapy, and department of hospitalization.

These decisions should be based on the basic principles of bioethics (beneficence, non-maleficence, autonomy, and justice), which means that we should choose the best option for the patient with no harm, considering and respecting his/her decision and with a correct distribution of resources. The balance between ageism and therapeutic futility is not easy. Ageism is any attitude, action, or institutional structure that subordinates a person or group exclusively based on age. It means stereotyping, prejudice, and discrimination against people based on their age [93]. A recent review showed that ageism is widespread and has a harmful effect on the health of older patients [94]. Moreover, physicians worry about how they will be treated when they are elderly, implying that they are aware of the ageism situation in clinical practice nowadays [95]. In patients with NSTEMI, we should not decide based only on age. A comprehensive geriatric evaluation that includes medical issues and comorbidity, mental status, social situation, and functional status (frailty and dependency) should be carried out [96]. This would help to establish prognosis and life expectancy and assess the utility or futility of treatment.

In older patients with NSTEMI, evidence is limited. As cardiac risk increases in the elderly, the absolute benefit of treatment should increase as well. However, the differences between trials and real-life patients may alter perceptions about the balance of risk and benefit derived from studies [97]. Clinical practical guidelines recommend applying the same diagnosis strategies as in younger patients, the same interventional strategies, and to adapt the choice of antithrombotic agent and dosage, as well as secondary preventions, to renal function and specific contraindications [13]. It is also recommended to assess ischemic and bleeding risks, estimated life expectancy, comorbidities, the need for non-cardiac surgery, quality of life, frailty, cognitive and functional status impairment, patients’ values and preferences, and the estimated risks and benefits of revascularization. If the patient is frail, the risk of individual treatment should be weighed up against the risk of harm. It would be reasonable to offer an invasive strategy to frail patients at high risk of cardiovascular events and low risk of complications, and to offer optimal medical therapy alone to those at low risk of future events with a high risk of developing procedural complications.

Although current indications are clear, some ethical conflicts could arise. As the coronavirus disease-19 sanitary emergency has shown, resources are limited. Thus, we must prioritize patients respecting the principle of justice, which states that equals should be treated equally. In patients with NSTEMI, we might have to choose the patient who is admitted to the cath lab or to an intensive care unit. Prioritization regulates the distribution of limited resources, and it does not mean that a person’s life is worth more than another, it means allocating the available resources in the most effective way and to those patients who are most likely to benefit (as we have always done in organ allocation) [98]. Age, per se, should not be a reason to prevent admission to intensive care unit or cath lab, and the social utility of every person simply for being human should be considered. Priority must be given to decisions that maximize survival to discharge and the number of life-years saved [99], but we should give patients a chance to live each stage of their lives to the fullest, admitting those patients who will benefit the most, independent of age or chronic diseases. Patients with a minimum expected benefit should not be admitted to the intensive care unit, and careful evaluation is required for those patients with reduced life expectancy [100].

## 8. Secondary Prevention in the Elderly

An individualized approach beyond age is essential when dealing with secondary prevention in older patients. A multidimensional assessment (comorbidity, functional, cognitive, social, nutritional, etc.) may guide a realistic adaptation of objectives. In a robust patient (i.e., independent, without relevant comorbidities or frailty), secondary prevention measures should be the same as in the general population. In a patient with severe or multiple comorbidities, the impact of these and its treatments should be weighted to adapt cardiovascular protective strategies. Finally, in severe frail and/or dependent patients, quality of life should be prioritized over survival, and lenient targets may be preferred.

Focusing on individual risk factors, dyslipidemia should follow the same recommendations as in their younger counterparts [101]. Statins are the drug of choice. However, evidence supporting high-dose statin therapy in older patients (especially over 85 years old) is scarce and inconclusive [102]. Afilalo et al. observed a significant decrease in mortality in elderly patients with coronary artery disease treated with statins, but LDL decreased less than 50% and mean levels at follow-up reached 90–100 mg/dL, challenging the need of high-intensity therapy [103]. A recent metanalysis including 14,483 patients > 75 years from 28 trials found that although statin therapy was associated with a decrease of major cardiovascular events, the benefit was smaller in older patients [104].

Hypertension is more severe and resistant to treatment and poses a higher risk of hypotension and falls in the older patient. Treatment should be carefully titrated because blood pressure control is associated with a reduction in cardiovascular events, but a strict control may have deleterious effects in this group of patients [105,106]. Similarly, diabetes treatment should avoid hypoglycemia, and therapeutic targets need to be modified according to patient status.

## 9. Current and Future Research Directions

There is still uncertainty about the optimal management of ACS in the older patient, and further research is warranted to clarify this issue. The SENIOR-RITA trial (NCT03052036), currently recruiting) is a randomized trial that aims to compare the invasive vs. conservative approach in ≥75 years old. Moreover, evidence is needed to address the role of frailty and geriatric syndromes in the management of ACS, such as the above-mentioned MOSCA-FRAIL trial.

## 10. Conclusions

ACS in the older patient constitute a challenge in clinical practice due to the peculiarities of this group, which pose a higher risk of both the disease and related to its therapeutic management. Moreover, specific evidence is scarce, and these patients are frequently undertreated. STEMI must be managed with urgent reperfusion following the general recommendations, and efforts should be made to avoid misdiagnosis and excessive delays. Therapeutic decisions in the older patient with NSTEMI should rely on a careful balance of risk and benefit, considering the comorbidities and the frailty beyond the age, and on the basis that invasive management offers clinical benefits. The bleeding risk may be higher in the elderly, and usual risk scores have shown a limited performance in this group of patients; thus, careful choice of antithrombotic treatment is warranted. A thorough assessment of the older patient must include frailty and other geriatric syndromes evaluation, being a cornerstone of comprehensive care in this setting.

## Figures and Tables

**Table 1:** A summary of some of the main evidence in ACS in the older patient.

Topic	Authors	Year	Study Type	Main Results
**Troponin in the elderly**	Boeddinghaus et al. [11]	2018	Observational	0/1 h troponin algorithm has similar rule-out safety but lower rule-in accuracy in ≥70 years. Specific thresholds may increase performance.
	Welsh et al. [8]	2018	Observational	Higher 99th percentile for troponin increases after the age of 60.
**NSTEMI**	Tegn et al. [32] (After-Eighty)	2016	Randomized trial	>80 years NSTEMI patients. Invasive vs. conservative strategy reduced combined endpoint of myocardial infarction, urgent revascularization, stroke, or death.
	Kaura et al. [33] (SENIOR-NSTEMI)	2020	Randomized trial	>80 years NSTEMI patients. Revascularization within the first 3 days of admission was associated to 32% reduction in all-cause mortality.
**Antithrombotic** **treatment**	Husted et al. [54] (PLATO substudy)	2012	Randomized trial	No increase in overall major bleeding with ticagrelor versus clopidogrel was observed in patients aged ≥ 75 years.
	Cayla et al. [57] (TRITON substudy)	2016	Randomized trial	No clinical benefit of prasugrel was observed in patients >75 years due to their higher rates of bleeding.
	Gimbel et al. [58] (POPULAR AGE)	2020	Randomized trial	>75 years NSTEMI patients. Lower bleeding rate with clopidogrel compared to ticagrelor or prasugrel, without an increase in the combined endpoint of all cause of death, myocardial infarction, stroke and bleeding.
**Geriatric** **conditions**	Llao et al. [81] (LONGEVO registry)	2018	Observational	Conservative treatment is associated with worse prognosis in older NSTEMI patients only if non-frail.
	Sanchis et al. [82] (MOSCA)	2016	Randomized trial	Older patient with comorbidities. Invasive strategy did not improve outcomes in terms of mortality or ischemic events at long-term follow-up.
	Sanchis et al.	2021	Randomized trial	Frailty status improved significantly after myocardial infarction if a cardiac rehabilitation program was followed.

NSTEMI: Non-ST-segment elevation myocardial infarction.
